# The patient experience of CHAPLE disease: results from interviews conducted as part of a clinical trial for an ultra-rare condition

**DOI:** 10.1186/s13023-024-03436-y

**Published:** 2025-02-11

**Authors:** Leighann Litcher-Kelly, Ahmet Ozen, Sarah Ollis, Hagit Baris Feldman, Andrew Yaworsky, Paolo Medrano, Voranush Chongsrisawat, Lorah Perlee, Marisa Walker, Sharanya Pradeep, Diane M. Turner-Bowker, Alina Kurolap, Orly Eshach Adiv, Michael J. Lenardo, Olivier A. Harari, Jessica J. Jalbert

**Affiliations:** 1 Adelphi Values, Boston, MA USA; 2 Marmara University, Istanbul, Türkiye; 3 Tel Aviv Sourasky Medical Center, Faculty of Medicine, Tel Aviv University, Tel Aviv, Israel; 4 Department of Pediatrics, Faculty of Medicine, Chulalongkorn University, King Chulalongkorn Memorial Hospital, Thai Red Cross Society, Patumwan, Bangkok, Thailand; 5 Regeneron Pharmaceuticals, Inc., Tarrytown, NY USA; 6 The Genetics Institute and Genomics Center, Tel Aviv Sourasky Medical Center, Tel Aviv, Israel; 7 Head of Pediatric Gastroenterology and Nutrition Unit, “Hillel Yaffe” Medical Center, Hadera, Israel; 8 Molecular Development of the Immune System Section, Laboratory of Immune System Biology, Laboratory of Clinical Immunology and Microbiology, and Clinical Genomics Program, National Institute of Allergy and Infectious Diseases, National Institutes of Health, Bethesda, MD USA

**Keywords:** CHAPLE, Within-trial interviews, Pozelimab, HRQoL

## Abstract

**Background:**

CD55 deficiency with hyper-activation of complement, angiopathic thrombosis, and protein-losing enteropathy (CHAPLE) disease is a newly identified condition with an estimated worldwide prevalence of < 100 patients. Patient interviews can ensure that what is important to patients is assessed in a clinical trial program. Due to the rare and potentially fatal nature of CHAPLE disease, interviews were conducted as part of the pozelimab clinical trial, rather than in a separate study before the trial. The aim of the interviews was to identify the key disease-related signs, symptoms, and health-related quality-of-life (HRQoL) impacts that are important and relevant to patients with CHAPLE disease.

**Methods:**

Interviews were conducted with patients and/or caregivers at two timepoints (screening and Week 24) during the pozelimab trial to document the signs/symptoms and HRQoL impacts of CHAPLE disease, and document the most bothersome sign/symptom at screening. At Week 24, interviews gathered additional information on the patient experience from caregivers and patients (note: the impact of pozelimab treatment was also collected, though these results are presented elsewhere).

**Results:**

Ten patients, aged 3–19 years, were enrolled in the trial; caregivers contributed to nine interviews. Thirty-one signs‌/symptoms and 65 HRQoL impacts were reported during the interviews. Abdominal pain, diarrhea, facial and peripheral edema/‌swelling, nausea, and vomiting emerged as the core signs/‌symptoms of CHAPLE disease (i.e., experienced by ≥ 90% of patients prior to treatment). The remaining 25 signs/symptoms were experienced by four or fewer (*n* ≤ 4, ≤ 40.0%) patients, and 15 were only reported by one patient each. Abdominal pain and facial edema were reported as the most bothersome signs/‌symptoms (*n* = 9, 90.0% and *n* = 1, 10.0%, respectively). The most frequently reported (i.e., ≥ 80% of interviews) HRQoL impacts were restricted diet (*n* = 10, 100.0%), sleep disruptions (*n* = 10, 100.0%), missing school (*n* = 9, 90.0%), ability to get dressed independently (*n* = 8, 80.0%), and difficulty engaging in play activities (*n* = 8, 80.0%).

**Conclusions:**

The main finding from these patient interviews is the identification of six core signs/symptoms of CHAPLE disease: abdominal pain, diarrhea, facial edema/swelling, peripheral edema/swelling, nausea, and vomiting. The severity of the core signs/symptoms leads to substantial impacts on patients’ lives.

**Trial registration:**

ClinicalTrials.gov, NCT04209634. Registered 20 December 2019 https://classic.clinicaltrials.gov/ct2/show/NCT04209634.

**Supplementary Information:**

The online version contains supplementary material available at 10.1186/s13023-024-03436-y.

## Introduction

Protein-losing enteropathy (PLE) is a term that describes a collection of conditions characterized by the excessive loss of serum proteins through the gastrointestinal tract. These conditions can arise as complications of primary gastrointestinal disorders or as secondary manifestations of systemic diseases [1]. PLE often presents with edema due to hypoalbuminemia, recurrent infections as a result of hypogammaglobulinemia, and a range of gastrointestinal symptoms. Nutrient malabsorption associated with PLE can lead to further complications. The wide variety of potential etiologies leads to complex symptomatology, underscoring the importance of precise symptom identification for accurate diagnosis, disease monitoring, and treatment evaluation.

CD55 deficiency with hyper-activation of complement, angiopathic thrombosis, and protein-losing enteropathy (CHAPLE) disease is an ultra-rare, potentially fatal condition caused by mutations in the *CD55* gene that prevent production of the CD55 protein that helps to regulate the complement system by accelerating the inactivation of the C3 and C5 convertases [1]. CHAPLE disease was first described in the literature in 2017 in two publications that described patients with PLE who were determined to have loss-of-function mutations in the *CD55* gene [1, 2]. Although PLE is the defining characteristic of CHAPLE disease, a substantial subset of patients suffer from grave complications, including thromboembolic events, which contribute to the condition’s high mortality rate [2]. While it is estimated that fewer than 100 patients worldwide have CHAPLE disease [3], the condition is associated with a high mortality rate [4–6], and until recently there were no approved treatments.

Pozelimab is a fully human, monoclonal immunoglobulin G4P antibody directed against the terminal complement protein C5, a key protein for activation of the terminal pathway of complement. Pozelimab is being evaluated in a clinical trial for patients ≥ 1 year old with CHAPLE disease (ClinicalTrials.gov: NCT04209634) [7] and was approved by the Food and Drug Administration (FDA) in August 2023 for the treatment of CHAPLE disease in patients 1 year or older [8, 9].

The primary efficacy results from the pozelimab clinical trial are presented elsewhere [9], and summarizes the pathophysiology of CHAPLE and the impact of pozelimab on the reduction in complement activation for patients with the condition. In planning for the pozelimab trial, it was known that pathogenic variations lead to primary intestinal lymphangiectasia and PLE, which could result in abdominal pain, chronic/recurrent diarrhea, vomiting, and edema that can be severe, requiring frequent hospitalizations and medical interventions, based on information from the literature and in discussions with physicians treating patients with the condition [1]. The available information demonstrated the highly symptomatic nature of CHAPLE disease; however, there were no published data describing the disease experience directly from the patient perspective. Best practices summarized in guidance documents published by the FDA [10–13] emphasize the importance of ensuring that the signs/‌symptoms/‌impacts that are most relevant from the patient perspective are assessed in the clinical trial. Due to the rare, severe, and potentially fatal nature of the disease, concept elicitation interviews were integrated within the conduct of the trial, rather than delaying the trial by first conducting a separate study to assess the patient experience of disease [14]. In this manuscript, the results of the within-trial interviews conducted to elucidate the patient experience of the signs, symptoms, and health-related quality of life (HRQoL) impacts of CHAPLE disease are reported.

## Methods

### Trial design

NCT04209634 is an ongoing, open-label, single-arm, multicenter, Phase 2/3 study evaluating the efficacy and safety of pozelimab in pediatric and adult patients with CHAPLE disease that will follow patients for up to 164 weeks [9]. This study is conducted in accordance with the principles of the Declaration of Helsinki and Council for International Organizations of Medical Sciences International Ethical Guidelines and was consistent with Good Clinical Practices of the International Conference on Harmonization and applicable regulatory requirements. Additional information about the trial methodology and primary results is presented elsewhere [9].

Along with input from clinicians with experience treating patients with CHAPLE disease, a review of the available CHAPLE disease literature was conducted to create a preliminary conceptual model to display the signs, symptoms, and HRQoL impacts of CHAPLE disease. This was subsequently reviewed by therapeutic area experts and revised based on their feedback. The conceptual model informed the clinical outcome assessment (COA) measurement strategy for the pozelimab clinical trial. The selection of the COAs to include in the clinical trial protocol considered the wide age range of patients enrolled in the clinical trial (i.e., babies to young adults), and the study team selected the PedsQL Core and Gastrointestinal Symptom modules [15–17] because of the different forms depending on the age of the patient (caregiver forms for the youngest patients, and different patient forms for school-age children, adolescents, and young adults); select COAs utilized within the clinical trial are included in Supplemental Table 1.

Interviews were conducted with each trial participant and/or caregiver (when applicable due to the patient’s age or cognitive ability) as part of the pozelimab clinical trial. The goal of the within-trial interviews was to confirm that the conceptual model and associated COA measurement strategy comprehensively captured the signs and symptoms that were important to patients with CHAPLE disease. In addition, following feedback from the FDA, the within-trial interview conducted before treatment at the screening visit included questions to document the most bothersome symptom (MBS) of CHAPLE disease from those concepts that were identified by clinical experts during the planning of the trial as being key to the condition (i.e., nausea, vomiting, stomach pain, diarrhea [frequent bowel movements and/or runny, loose, watery poop], swelling in the face, and swelling in the arms and legs). The MBS was included as a primary endpoint in the study protocol to capture clinical benefit from the patient perspective. Specifically, the MBS was documented to evaluate how the experience of the patients’ MBS changed pre- to post-treatment.

### Interview methods

Interviews were conducted at two timepoints during the clinical trial, at screening and at Week 24 (W24), the timepoint for the evaluation of the primary endpoint. Semi-structured 60-minute interviews were conducted by trained interviewers with all trial participants and/or their caregivers. Each interview was conducted in person or via telephone (an option for the W24 interviews due to the COVID-19 pandemic) in the native language of the participant, audio-recorded, transcribed, anonymized, and translated into United States (US) English by an independent transcription company.

Patients 8 years of age or older were the primary respondent of the interview, with input from their caregiver, as appropriate; for patients younger than 8 years old, or those with cognitive impairments, the caregiver was the primary or sole respondent of the interview, although the patient was also invited to contribute. The screening interviews aimed to identify the signs, symptoms, and HRQoL impacts of CHAPLE disease that were important and relevant to the trial participants before starting the study treatment, and also to document the MBS. Patients and caregivers were asked to provide ratings of severity, bothersomeness, and impact of the following signs/symptoms (if the patient reported experiencing them): abdominal pain, diarrhea, facial edema, nausea, peripheral edema, and vomiting. Respondents used a 0–10 numeric rating scale (NRS), provided verbally, where 0 was not severe/bothersome/impactful and 10 was extremely severe/bothersome/impactful.

While information from the W24 interviews focused on the change experienced in the signs, symptoms, and HRQoL impacts after 6 months of pozelimab treatment (results described elsewhere [18]), additional sign, symptom, and impact concepts were collected during these interviews, as some concepts were not originally reported during the screening interviews. These concepts were included to better describe and capture the full patient experience of CHAPLE disease.

### Analysis

Transcripts were anonymized by removing any potentially identifying information (e.g., names of people, places) from the transcripts.

**Fig. 1:**
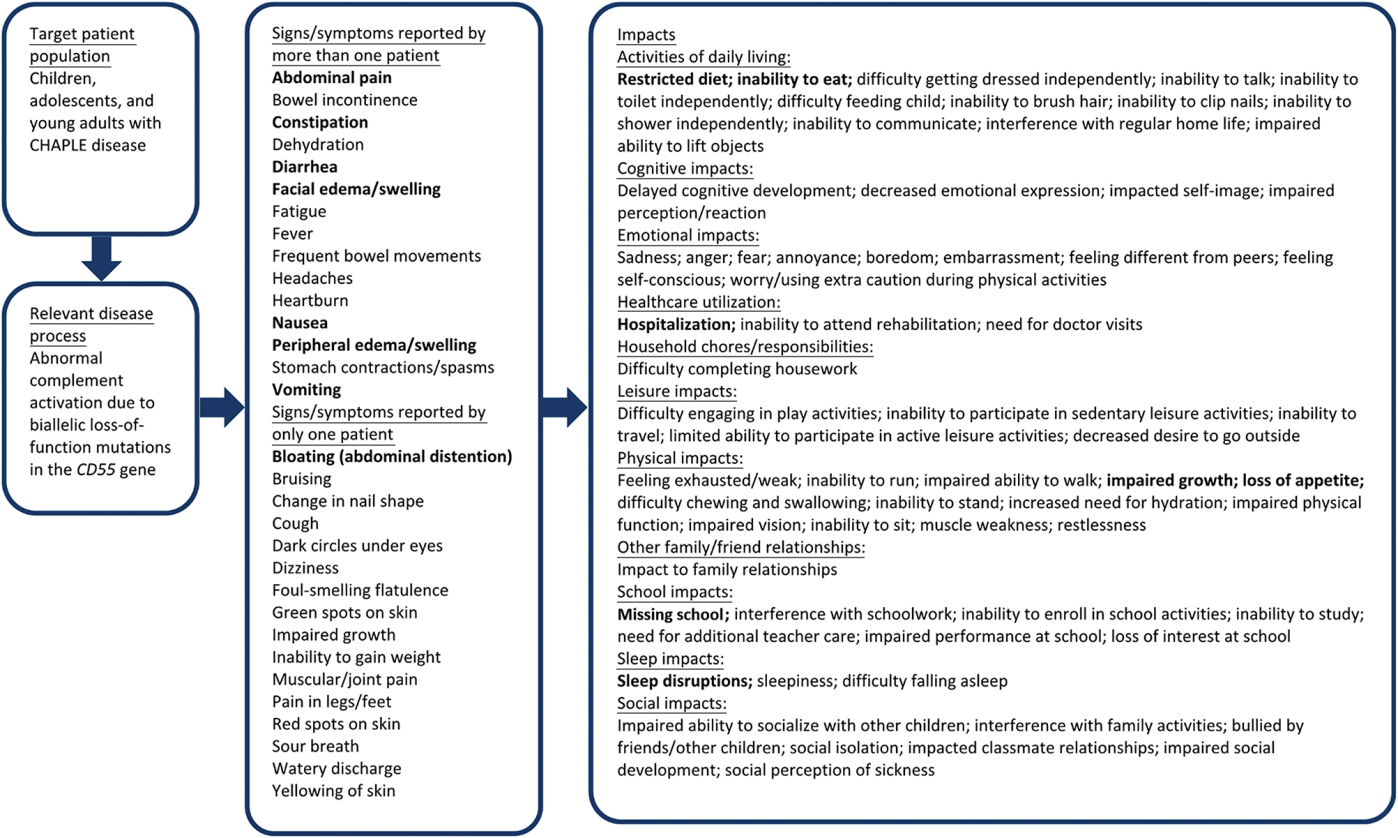
CHAPLE sign, symptom, and impact conceptual model: screening and W24. Bolded concepts were reported by both clinical experts and by patients/caregivers

Interview data elicited from patients and caregivers from the screening and W24 interviews pertaining to the disease experience were coded for each patient. Coding and analysis were performed on the translated transcripts, using qualitative data analytic methods, following a pre-specified Qualitative Data Analysis Plan. The primary goal of coding was to organize and catalog participants’ descriptions of CHAPLE disease-related signs, symptoms, and HRQoL impacts to develop a patient-centric conceptual model (Fig. 1). Following analysis, the interview data from patients and caregivers were pooled and used to calculate concept frequency, conduct concept description analysis, and develop concept characterizations (i.e., concept descriptions from patient/‌caregiver descriptions). Following best practices [12], HRQoL impacts were grouped into conceptual domains iteratively, first based on a preliminary conceptual framework and then updated based on participant quotes. Additionally, ratings for severity, bother, and impact were collected for the core signs/symptoms only, if time during the interview allowed.

## Results

### Patient characteristics

Ten patients were enrolled in the clinical trial at sites in three countries: Türkiye (*n* = 7), Thailand (*n* = 2), and the US (*n* = 1). The mean age of patients was 9.3 years (standard deviation [SD] = 4.9), and more than half were female (*n* = 6; 60.0%). One young adult patient completed both the screening and W24 interviews independently, while the remaining interviews were conducted either as dyads (*n* = 5 at screening and *n* = 6 at W24) or with caregiver only (*n* = 4 at screening and *n* = 3 at W24), due to the age or ability of the patient. Table 1 summarizes the demographic information of the sample.

**Table 1:** Site-reported patient demographic information (*N* = 10)

Characteristic	Total (*N* = 10) *n* (%)
**Age (years)**	
Median (range)	8.5 (3–19)
Mean (standard deviation)	9.3 (4.9)
**Age groups** ^*^	
≤ 7 years of age	4 (40.0%)
≥ 8 to < 12 years of age	4 (40.0%)
≥ 12 to < 18 years of age	1 (10.0%)
≥ 18 years of age	1 (10.0%)
**Sex**	
Female	6 (60.0%)
Male	4 (40.0%)
**Interview participants (Screening/W24)** ^**†**^	
Patient only	1 (10.0%)/1 (10.0%)
Patient/caregiver dyad	5 (50.0%)/6 (60.0%)
Caregiver only	4 (40.0%)/3 (30.0%)
**Country of origin (native language)** ^**‡**^	
Bolivia (Spanish)	1 (10.0%)
Syria (Arabic)	2 (20.0%)
Thailand (Thai)	2 (20.0%)
Türkiye (Turkish)	5 (50.0%)

^*^Patients ≥ 12 years old completed COAs; caregivers of patients < 12 years old completed COAs

^†^Generally, patients aged ≥ 8 years with or without their caregivers were the primary respondent for interviews, and for patients < 8 years caregivers were the primary respondents

^‡^While most patients (*n* = 7, 70.0%) received treatment in their native country, the patients from Bolivia (*n* = 1, 10.0%) and Syria (*n* = 2, 20.0%) were treated in the US and Türkiye, respectively

### Signs and symptoms

A total of 31 signs and symptoms were reported by participants during the screening and/or W24 interviews as part of their pre-treatment disease experience [19] (Supplemental Table 2). Abdominal pain, facial edema/‌swelling, peripheral edema/swelling, diarrhea, and vomiting were reported to be experienced by all patients (*n* = 10, 100.0%) and nausea was reported to be experienced by all but one patient (*n* = 9, 90.0%). These concepts, reported to be experienced by ≥ 90.0% of patients, were considered to be the core signs and symptoms of the disease. There were an additional 25 signs/symptoms reported by four or fewer (*n* ≤ 4, ≤ 40.0%) patients, with most (15 signs/symptoms) reported idiosyncratically by only one patient (Supplemental Table 2).

Abdominal pain was reported to be the MBS for all but one patient (*n* = 9, 90.0%); for the final patient (*n* = 1, 10.0%) facial edema/swelling was reported to be the most bothersome. Two participants also reported a secondary MBS. Vomiting (*n* = 1, 10.0%) and headaches (*n* = 1, 10.0%) were also reported to be most bothersome to patients secondary to abdominal pain. Abdominal pain and facial edema/swelling were the only signs and symptoms to be reported as the most important to improve with treatment (*n* = 7, 70.0% and *n* = 2, 20.0%, respectively).

Note that the interviews with caregivers included follow-up questions to understand whether and how caregivers were able to report on the presence of symptoms, which are generally considered to be only reportable by the patients themselves. Caregivers described their observations of patient behavior (e.g., for abdominal pain, crying, holding one’s belly, and screaming were described), being expressly told by the patient, and/or by being able to see or feel indications that a symptom was occurring (e.g., caregivers describe indications of abdominal pain as “round and hard stomach” and “lower abdomen hard as a stone”). Thus, caregiver-reported data were retained to contextualize the patient’s disease experience. Severity, bother, and impact of the core signs and symptoms were assessed using a 0–10 NRS, from the patient and/or caregiver perspective. Due to the conversational nature of the interviews, not all patients/caregivers were asked to provide all ratings for these six signs and symptoms. Abdominal pain had the highest median severity, bother, and impact scores from both the patient and caregiver perspective (see Supplemental Table 3 for all available ratings for the six core signs and symptoms). Exemplary quotes for the severity of the six core signs/symptoms are presented in Table 2 to provide additional context for the severity of these signs/symptoms.

**Table 2:** Exemplary quotes from patients and caregivers about each CHAPLE disease core sign and symptom

Symptom	*n* (%)	Exemplary quotes
Abdominal pain	10 (100.0%)	*Exemplary quote 1*: **PATIENT**: If my abdominal pain is severe, I even cannot get up. I literally cannot get up if I do not have someone next to me to take my arm and help me in getting up. I started experiencing this more often for a few years, three, even four years. Previously, I was able to handle it somehow when I had pain, good or bad, but for a couple of years at least, the situation is not like that. It started to be severer and make me feel more exhausted. After I have abdominal pain, I feel drowsy. That day is literally wasted. It is very rare that I can continue my day. *Exemplary quote 2*: **CAREGIVER**: Stomach pain – first, her stomach will swell, like she is bloated. It gets round and hard, so we can feel her stomach and that its contracted. We can feel it. Then, she’ll have a twisting pain, and she’ll cry out – like, it’s very painful. If there’s a full score, I’d give it the full score.
Diarrhea	10 (100.0%)	*Exemplary quote 1*: INTERVIEWER: OK. And what was the main reason why you went to the doctor’s? **PATIENT**: Lots of diarrhea, vomiting, and finally, I started having stomachaches. I got very dehydrated and they took me (to the hospital) and hooked me to a saline drip, painkillers and sent me back home. At [REDACTED] all they did was give me a saline drip and painkillers, and they would send me back home. They didn’t prescribe any treatment.… INTERVIEWER: First, let’s talk about the symptoms you have due to your disease. You told me that it started with diarrhea. PATIENT: Diarrhea, vomiting, stomachache. Those were the three main symptoms I had; I didn’t get – Sometimes I had a fever, but rarely. When I was vomiting a lot or having diarrhea, I felt weak. *Exemplary quote 2*: INTERVIEWER: How often does she get diarrhea? How many times per month? How many times per week? Let’s understand that first and then we can go into details. **CAREGIVER**: Sometimes it lasts 3 days or 4 days per week.… Diarrhea has never stopped on its own. If I give her potatoes. Sometimes it does not stop at all and I take her to the hospital. INTERVIEWER: Does she get an IV line at the hospital? CAREGIVER: Yes. I mean, here is the thing. Even though I give her potatoes, it is not resolved completely, it just softens. Sometimes, I give her yoghurt. INTERVIEWER: Do they have to go to hospital? CAREGIVER: She definitely goes once per month due to diarrhea.… INTERVIEWER: Okay. How bad is diarrhea? CAREGIVER: [SPEAKING ARABIC] That is 9 as well, because her whole body shakes after she gets up from the toilet seat.
Facial edema/‌swelling	10 (100.0%)	*Exemplary quote 1*: **PATIENT**: Swelling in the face.… INTERVIEWER: Yes, you said swelling in the eyes and face; I am asking about that. How much pain did you have? PATIENT: The pain was as if I had been sting.… The swelling was too much here. *Exemplary quote 2*: INTERVIEWER: Let’s talk about your child’s stomach and eyes again, how bad do you think the swelling in the eye is for your child? **CAREGIVER**: Ten. INTERVIEWER: Why? CAREGIVER: He could not see anything. He could not open his eye. I mean, his eyes were excessively swollen. He could not open his eyes either. Therefore, he could not see anything.
Nausea	9 (90.0%)	*Exemplary quote 1*: **PATIENT**: Five. INTERVIEWER: Five? Why nausea and why did you choose five? PATIENT: Because I feel bad when I have nausea. INTERVIEWER: What does it prevent? PATIENT: When I play with the ball or go out and run, I have nausea and sometimes I have heartache. Therefore, I stop and I felt like that before. I do not want to vomit. *Exemplary quote 2*: INTERVIEWER: Does she get nausea? **CAREGIVER**: [SPEAKING ARABIC] Yes. INTERVIEWER: Alright, does she vomit when she gets nausea? CAREGIVER: [SPEAKING ARABIC] She vomits every time she feels nauseous. INTERVIEWER: Alright, then I will ask for the same detailed explanation with this one as well, also tell me that. How does she understand that the child gets nausea? … Does the child tell her or does she realize it? CAREGIVER: [SPEAKING ARABIC] No, she tells me herself. Or she makes sounds like ughhh and that is how I understand it. Sometimes, she cannot wait to tell me. INTERVIEWER: Does she run to the bathroom immediately? CAREGIVER: [SPEAKING ARABIC] Sometimes I don’t see her at all, she just throws up, I mean she lets it out. INTERVIEWER: Alright, did the child tell her or explain now or in the past how nausea or vomiting made her feel? CAREGIVER: [SPEAKING ARABIC] She says, Mom, I feel relieved when I throw up. INTERVIEWER: So does she actually throw up to feel relieved? CAREGIVER: [SPEAKING ARABIC] Yes.
Peripheral edema/‌swelling	9 (90.0%)^*^	*Exemplary quote 1*: INTERVIEWER: Okay, swelling in the face. I asked about your life, I’m sorry. We did not ask about the swelling in your arms and legs. How many points? **PATIENT**: Seven. INTERVIEWER: Seven. Why did you give seven points; tell me about it as well? PATIENT: Because I feel uncomfortable when my feet and my hands become swollen. I feel uncomfortable. *Exemplary quote 2*: INTERVIEWER: How long would it last? Swelling in the hands-feet? **CAREGIVER**: It lasts for 1–2 days, then it goes away. But it continues in the eyes.… INTERVIEWER: How long does this swelling last? Doesn’t it go away until she takes the medicine or otherwise? CAREGIVER: It gets resolved after she takes the medicine, 2–3 days before she takes the medicine, this Solaris as I said, her swelling and loss of appetite complaints start again. Vomiting starts.
Vomiting	10 (100.0%)	*Exemplary quote 1*: INTERVIEWER: Vomiting, now, how does vomiting make you feel? What can you tell? How does it happen? **PATIENT**: I mean it causes exhaustion. I do not know if anything diminishes. Because vomiting is sometimes excessive. INTERVIEWER: How is it? How much? PATIENT: I mean, I feel like nothing is left in my stomach. I get it; we feel like that when we are hungry. I literally feel like that. I sometimes drink some water and throw it up after one-two minute(s). It happens like that. I mean, it is a bit challenging in that aspect. *Exemplary quote 2*: **CAREGIVER**: For example, children eat something once every hour, I try to make her eat once every 3 hours. I mean, she is not a child who eats non-stop. I mean, as she is vomiting all the time now, her muscle development is – I had to give her formula all the time from the hospital.

^*^One participant who did not report peripheral edema at screening indicated at W24 that they did experience peripheral edema before starting the study medication

**Table 3:** Exemplary quotes for most frequently reported impacts of CHAPLE disease

Impact	*n* (%)	Exemplary quotes
Restricted diet	10 (100.0%)	**CAREGIVER**: When socializing, she can’t eat what other people are eating.… It’s like she’s not like other children who can live in society.… when other people’s children go out to eat, they don’t need to do anything, but our kid can’t eat like everyone else’s kids. She can only sit and watch.…
Sleep disruption	10 (100.0%)	**PATIENT**: I can’t sleep well; those nights when I’m in pain I don’t sleep at all; it hurts all the time; last night I was lying there from 10 pm until 1:30am… I was crying.
Missing school	9 (90.0%)	**CAREGIVER**: She misses school very often. In a week, she barely goes to school.… Even when it’s the semester break, she’ll have to go back to school to take make-up exams because she hasn’t taken them yet.
Difficulty engaging in play activities	8 (80.0%)	**CAREGIVER**: She cannot play, she walks around and cries. She cries all the time. Sometimes she gets upset and pulls her hair …
Difficulty getting dressed independently	8 (80.0%)	**CAREGIVER**: She cannot get dressed on her own. INTERVIEWER: Why? CAREGIVER: I mean she cannot get dressed because she cannot close it on her stomach. She cannot wear a skirt, because she cannot button it on her stomach…. For example, she cannot wear her boots, which is a very simple thing. INTERVIEWER: She cannot bend down. CAREGIVER: She cannot bend down, she cannot wear them.… I mean, she cannot get dressed in general.

### Health-related quality-of-life impacts

A total of 65 HRQoL impacts were reported by participants during the screening and/or W24 interviews (Supplemental Table 4). The HRQoL impacts were reported across 11 domains: activities of daily living (ADL), cognitive impacts, emotional impacts, healthcare utilization, household chores/responsibilities, leisure impacts, physical impacts, other family/friend relationships, school impacts, sleep impacts, and social impacts. The domains with the most reported impacts were physical impacts (*n* = 13 impact concepts), ADL (*n* = 12 impact concepts), and emotional impacts (*n* = 9 impact concepts). The most frequently reported impact concepts (≥ 80% of patients) across both interviews were: restricted diet (*n* = 10, 100.0%; ADL domain), sleep disruptions (*n* = 10, 100.0%; sleep domain), missing school (*n* = 9, 90.0%; school domain), getting dressed independently (*n* = 8, 80.0%; ADL domain), and difficulty engaging in play activities (*n* = 8, 80.0%; leisure domain). Exemplary quotes for the most frequently reported impacts are presented in Table 2.

### CHAPLE conceptual model

The final conceptual model, inclusive of all signs, symptoms, and impacts reported at screening and W24, is presented in Fig. 1. Concepts discussed by therapeutic experts are bolded.

## Discussion

The key finding from this study is the identification of six core signs and symptoms of CHAPLE disease: abdominal pain, diarrhea, facial edema/swelling, peripheral edema/swelling, nausea, and vomiting. Abdominal pain was reported to be the MBS for 90.0% of patients and identified as the most important to improve with treatment. Abdominal pain was also consistently identified as the most severe, bothersome, and impactful of the signs and symptoms reported by patients with CHAPLE disease and their caregivers. In addition to the six core signs and symptoms, other signs and symptoms were reported, but most were only reported by one patient each. Therefore, CHAPLE disease is characterized by six core signs and symptoms, which, from the patient perspective, may suggest a homogeneous disease presentation. The impacts reported spanned eleven domains, with the most frequently reported impacts in the ADL, sleep, school, and leisure domains. The results from these within-trial interviews demonstrate that the CHAPLE disease experience, inclusive of the six core signs and symptoms, impacts patients’ lives, especially diet, sleep, school, and play. These patient-centric results build on the prior work to describe this newly identified disease [1, 2, 4–6].

Given the nature of conducting clinical trials in an ultra-rare, newly described fatal disease, the COA measurement-strategy [10–13] was based on information from the available literature and data from discussions with therapeutic area experts/treating clinicians, and the goal of the within-trial interviews was to confirm that the important signs/‌symptoms and HRQoL impacts were assessed by the selected COAs and to ensure that no significant sign and symptom concepts were left out. Since there was the potential for a heterogeneous phenotype and the small trial sample, the FDA guided the clinical trial development team to document each patient’s MBS, and these data were included as a key endpoint for the clinical trial. The results of the within-trial interviews indicated that the MBS for nearly all patients in the trial was abdominal pain. The results from the interviews, specifically the MBS results, confirmed the comprehensiveness of the COA measurement strategy. Further, during the W24 within-trial interviews, patients reported a complete resolution of the core CHAPLE-related signs and symptoms they had experienced. These results aligned with the COA measurement strategy, results of which are described elsewhere [18]. Within-trial interview data provided supportive evidence of the treatment benefit of pozelimab for CHAPLE disease from the perspective of patients with CHAPLE disease and their caregivers. The data from the within-trial interviews allowed for a more complete picture of the CHAPLE disease experience and contributed to the totality of evidence regarding the treatment benefit of pozelimab.

The results of this study must be interpreted in the context of certain limitations. Not all patients could be the primary respondent due to their ages, so caregivers provided data on their child’s experience of the signs and symptoms of disease as a proxy report. However, during interviews, caregivers were asked to describe and were able to explain how they were aware of the symptoms their child was experiencing and their severity, and they reported both verbal and nonverbal ways their child communicated their symptoms from the lens of observers [19]. Further, this study was conducted with a small sample size of ten participants, in three countries, and was not a randomized controlled trial, as all participants received treatment with pozelimab due to the ultra-rare, life-threatening nature of CHAPLE disease.

## Conclusion

Once the disease manifests with active symptoms, CHAPLE disease is a rare condition, with risk of fatality, that includes a core set of severe signs and symptoms (abdominal pain, diarrhea, facial edema/swelling, peripheral edema/swelling, nausea, and vomiting) that have significant and broad impacts on patients’ day-to-day life. Findings from the within-trial interviews were helpful in elucidating the patient experience of the signs, symptoms, and HRQoL impacts of this newly identified ultra rare disease. Within-trial concept elicitation interviews may be helpful to conduct when the disease is potentially fatal, ultra-rare, and has an unmet need for treatment. The data from the within-trial interviews conducted as part of the clinical trial provide support for the treatment benefit of pozelimab. Broadly, within-trial interviews may be helpful for the elucidation of a more robust description of ultra-rare, fatal diseases without existing treatment.

## Electronic supplementary material

Below is the link to the electronic supplementary material.

Supplementary Material 1

## Data Availability

Qualified researchers may request access to study documents (including the clinical study report, study protocol with any amendments, blank case report form, and statistical analysis plan) that support the methods and findings reported in this manuscript. Individual anonymized participant data will be considered for sharing once the product and indication has been approved by major health authorities (e.g., US Food and Drug Administration, European Medicines Agency, Pharmaceuticals and Medical Devices Agency, etc.), if there is legal authority to share the data and there is not a reasonable likelihood of participant re-identification. Requests should be submitted to https://vivli.org/.
