# Supplementary material for: The patient experience of CHAPLE disease: results from interviews conducted as part of a clinical trial for an ultra-rare condition
Source: Orphanet J Rare Dis. 2025 Feb 11;20:68. doi: 10.1186/s13023-024-03436-y (PMC11817392; doi:10.1186/s13023-024-03436-y)
Supplement: Supplementary file 1 — Supplementary Material 1 [file 13023_2024_3436_MOESM1_ESM.docx]

Supplemental tables

| Supplemental table 1. Select clinical outcomes assessments utilized in the pozelimab clinical trial | |
| --- | --- |
| Core sign and symptom concepts | Clinical outcome assessment (COA) |
| Abdominal pain | PedsQL™ GI Symptoms (stomach pain and hurt) subscale |
| Diarrhea | PedsQL™ GI Symptoms (diarrhea) subscale |
| Facial Edema | Clinical assessment of facial edema |
| Peripheral Edema | Clinical assessment of peripheral edema |
| Nausea and vomiting | PedsQL™ GI Symptoms (nausea and vomiting) subscale |

| Supplemental table 2 CHAPLE sign and symptom description table (N=10) | | |
| --- | --- | --- |
| Concept reported by study participant | Concept description based on reports by study participants at Screening or W24 interview | Overall participant report  n (%) |
| Abdominal pain | Described by patients and caregivers as hurting, aching, tightness, straining, and/or cramping in the stomach and/or abdomen that may be accompanied by nausea, vomiting, and diarrhea,  Caregivers describe pain behaviors, such as crying and holding one’s belly, while abdominal pain occurs | 10 (100.0%) |
| Diarrhea | Described by patients as a bowel movement with a watery or soft consistency, which may be accompanied by abdominal pain, urgency, and/or frequent need to go. Reported bowel movement colors varied (e.g., orange, brown, yellow), and one patient attributed the symptom as a contributor to feelings of weakness.  Caregivers describe watery, liquid, or soft stool. | 10 (100.0%) |
| Facial edema/‌swelling | Described by patients as the face and/or eyelids becoming swollen, causing pain (similar to a bee sting)  Caregivers as the face, areas around the eye or eyelids, forehead, cheeks, and/or nose becoming swollen (due to fluid retention); also associated this with pain (similar to a bee sting) | 10 (100.0%) |
| Vomiting | Described by patients and caregivers as throwing up stomach fluids due to physical activity and/or eating food/drinking; often associated with nausea. A patient noted that this condition was associated with their experience of sour breath | 10 (100.0%) |
| Peripheral edema/‌swelling | Described by patients and caregivers as fluid retention, or fluid in the body (e.g., hands, feet, legs, arms, etc.) that leads to swelling; can also cause pain | 10 (100.0%) |
| Nausea | Described by patients and caregivers as feeling the urge to vomit or feeling sick, which can occur with vomiting and stomach/abdominal pain  Caregivers described the patient communicating their feeling of nausea or wanting to vomit. Caregivers also reported observing the patient being nauseous with sounds (“she makes sounds like ughhh and that is how I understand it.”), facial expressions, or actions (“She makes a sour face, you know. Sometimes she would try to vomit even though she did not eat anything”) | 9 (90.0%) |
| Fever | Described by a patient as a lesser symptom of enteropathy that has occurred without infection  Caregivers describe as the body being hotter than normal due to disease; may be accompanied by vomiting and/or diarrhea; may be caused by infection | 4 (40.0%) |
| Bloating | A patient described bloating as pain, aches, cramps, and hurt in the stomach area | 3 (30.0%) |
| Bowel incontinence | A patient described bowel incontinence as instances in which they may have an accident while sleeping  Caregivers describe as having diarrhea or not getting to the restroom in time (which could lead to the patient having an accident in their pants) | 3 (30.0%) |
| Headaches | Described by patients as pain in/around the head that occurs when there is swelling and low albumin levels  Caregivers describe pain behaviors such as holding or pulling hair and squirming. | 3 (30.0%) |
| Frequent bowel movements^*^ | Patients described frequent passing stool as liquid, water-like stool passing frequently | 3 (30.0%) |
| Constipation | A patient described constipation as the inability to pass bowel movement despite feeling the urge to defecate  Caregivers described constipation as the inability to have a bowel movement regularly, causing pain and the lower abdomen to feel “like a stone” | 2 (20.0%) |
| Dehydration | A patient described having issues with staying hydrated as something that requires them to be given a saline drip at the hospital and perceived to be triggered by vomiting and diarrhea  A caregiver described one afflicted child as appearing dehydrated and having sunken eyes | 2 (20.0%) |
| Fatigue | Caregivers described fatigue as being weak and easily tired/‌exhausted when engaging in physical activities | 2 (20.0%) |
| Heartburn | A patient described heartburn as feeling like throwing up and having convulsions, contractions, and pain  A caregiver described heartburn as getting an uncomfortable feeling and feeling like throwing up | 2 (20.0%) |
| Stomach contractions/‌spasms | A patient described stomach contractions/spasms as being a prominent symptom that feels like contractions and that can occur with heartburn  A caregiver described stomach contractions/‌spasms as a twisting feeling in the intestines that sends a wave through the abdomen | 2 (20.0%) |
| Bruising | A caregiver described bruising as marks on the body, specifically on the abdomen and face | 1 (10.0%) |
| Change in nail shape | A caregiver described change in nail shape once the patient was two to three years old | 1 (10.0%) |
| Dark circles under eyes | A caregiver described their child developing dark circles under their eyes along with eye swelling | 1 (10.0%) |
| Foul-smelling flatulence | A caregiver described foul-smelling flatulence as occurring as a result of enteritis | 1 (10.0%) |
| Green spots on skin | A caregiver described green spots as spots that might be related to cancer and occur for two to three days, then disappear | 1 (10.0%) |
| Inability to gain weight | No description was provided by the patient regarding the inability to gain weight | 1 (10.0%) |
| Red spots on skin | A caregiver described red spots occurring when the patient was not on treatment; the spots occur with swelling, loss of appetite, and vomiting | 1 (10.0%) |
| Sour breath | A patient described sour breath as occurring prior to vomiting due to abdominal pain | 1 (10.0%) |
| Watery discharge | A caregiver described water discharge as water coming out of the patient’s face and veins instead of blood | 1 (10.0%) |
| Yellowing of skin | A caregiver described yellowing of skin as occurring in tandem with a change of mood, reporting that the patient changes color and turns yellow | 1 (10.0%) |
| Cough^*^ | Caregivers described cough as having a continuous cough as a result of the disease | 1 (10.0%) |
| Dizziness^*^ | One patient mentioned that dizziness was a symptom of the disease, but provided no additional context about their dizziness or when it occurs | 1 (10.0%) |
| Impaired growth^*^ | Impaired growth was described as children being smaller and paler as a result of being unable to eat as much | 1 (10.0%) |
| Muscular/joint pain^*^ | Muscular/joint pain was described as a symptom due to CHAPLE disease. No other context was provided around the muscular/joint pain by the patient | 1 (10.0%) |
| Pain in legs/feet^*^ | Pain in legs and feet was described as pain that occurred in the feet and legs when walking, which made running not possible. After treatment, the participant reports being able to walk long distances in short periods of time | 1 (10.0%) |

^*^Six symptoms were not reported at screening but were reported at W24 to have been experienced by patients **prior to treatment**.

| Supplemental table 3. Screening interview bother, severity, and impact ratings table for core signs and symptoms | | | |
| --- | --- | --- | --- |
| Concept  (n) | Aspect^*^ by respondent | Pre-treatment (CE/Screening) | |
|  |  | Participants providing rating  n | Median (Min–Max)^†^ |
| Abdominal pain  (n=10) | Patient-self report (N=6) | | |
|  | Severity | 4 | 9 (8–10) |
|  | Bother | 5 | 9 (8–10) |
|  | Impact | 4 | 9.5 (8–10) |
|  | Caregiver report (N=9) | | |
|  | Severity | 4 | 9.5 (8–10) |
|  | Bother | 5 | 10 (9–10) |
|  | Impact | 4 | 10 (10–10) |
| Facial edema/swelling  (n=10) | Patient-self report (N=6) | | |
|  | Severity | 1 | 8 (8–8) |
|  | Bother | 5 | 5 (2–9) |
|  | Impact | 2 | 6.5 (3–10) |
|  | Caregiver report (N=9) | | |
|  | Severity | 3 | 5 (2–10) |
|  | Bother | 5 | 8 (7–10) |
|  | Impact | 3 | 2 (0–10) |
| Diarrhea  (n=10) | Patient-self report (N=6) | | |
|  | Severity | 2 | 5.5 (4–7) |
|  | Bother | 5 | 5 (3–7) |
|  | Impact | 3 | 7 (4–8) |
|  | Caregiver report (N=9) | | |
|  | Severity | 3 | 10 (9–10) |
|  | Bother | 5 | 8 (0–10) |
|  | Impact | 3 | 10 (9–10) |
| Peripheral edema/‌swelling  (n=9)^‡^ | Patient-self report (N=6) | | |
|  | Severity | 2 | 5.5 (4–7) |
|  | Bother | 4 | 5 (2–6) |
|  | Impact | 2 | 5 (3–7) |
|  | Caregiver report (N=9) | | |
|  | Severity | 2 | 1.5 (1–2) |
|  | Bother | 5 | 2 (0–10) |
|  | Impact | 2 | 1 (0–2) |
| Vomiting  (n=10) | Patient-self report (N=6) | | |
|  | Severity | 3 | 5 (4–7) |
|  | Bother | 5 | 7 (3–10) |
|  | Impact | 3 | 6 (4–7) |
|  | Caregiver report (N=9) | | |
|  | Severity | 2 | 9.5 (9–10) |
|  | Bother | 5 | 8 (5–10) |
|  | Impact | 3 | 10 (9–10) |
| Nausea  (n=9) | Patient-self report (N=6) | | |
|  | Severity | 3 | 5 (5–7) |
|  | Bother | 4 | 5.5 (5–8) |
|  | Impact | 3 | 5 (5–8) |
|  | Caregiver report (N=9) | | |
|  | Severity | 3 | 9 (8–10) |
|  | Bother | 4 | 9.5 (8–10) |
|  | Impact | 3 | 8 (8–10) |

^*^0–10 NRS where higher scores indicate a higher level of severity, bother, or impact

^†^Medians that are not whole numbers were due to an even number of ratings available

^‡^One participant who did not report peripheral edema at Screening, indicated at W24 that they did experience peripheral edema before starting the study medication.

| Supplemental table 4. CHAPLE impact description table (N=10) | | |
| --- | --- | --- |
| Concept reported by study participant | Concept description based on reports by study participants at Screening or W24 interview | Overall participant report  n (%) |
| Activities of daily living | | |
| Restricted diet | Described by patients described as the inability to eat certain foods (e.g., apricots, figs, junk food, sweets, fried food, canned food, soda) in order to avoid worsening the condition  Described by caregivers as providing certain foods (e.g., potatoes, boiled rice) to avoid or relieve symptoms, having restrictions on what their child could eat, or avoiding food in general in order to prevent symptoms | 10 (100.0%) |
| Inability to eat | A patient described inability to eat as vomiting up anything they ate for a period of one to two weeks  Described by caregivers as the child’s inability to drink water, removing food inserted in their mouth, vomiting up even small amounts of food immediately, or stomach pain making it “almost impossible” to eat | 5 (50.0%) |
| Difficulty getting dressed independently | Described by caregivers as needing to assist in putting clothing on due to the child’s swelling interfering with the process, or the child being unable to get dressed | 8 (80.0%) |
| Inability to talk | Described by caregivers as the inability to speak due to pain or due to physical or cognitive underdevelopment | 2 (20.0%) |
| Inability to toilet independently | Described by caregivers described as needing to always be with the child when they have diarrhea, or as the child’s lack of toilet training due to delayed development | 2 (20.0%) |
| Difficulty feeding child | A caregiver described difficulty feeding as the child’s inability to eat by themselves | 1 (10.0%) |
| Inability to brush hair | A caregiver described their child’s inability to brush their own hair due to illness | 1 (10.0%) |
| Inability to clip nails | Described by a caregiver as their child’s inability to cut their own nails due to the child feeling sluggish and tired due to the disease | 1 (10.0%) |
| Inability to shower independently | A caregiver stated that their child was unable to shower and help themselves when experiencing symptoms | 5 (50.0%) |
| Inability to communicate^*^ | Described after treatment as the ability to communicate (e.g., talk and learn new words) with siblings and other family members in an effective manner in order to meet their needs | 1 (10.0%) |
| Interference with regular home life | A caregiver described interference with regular home life as being unable to do regular activities such as having breakfast, listening to music on television, and generally being together as a family due to hospital trips for the affected child | 1 (10.0%) |
| Impaired ability to lift objects^*^ | Described after treatment as the ability to lift weight between 2–5 kg and assist others by carrying items | 2 (20.0%) |
| Cognitive impacts | | |
| Delayed cognitive development | Described by caregivers as the child being behind their peers’ development in areas such as walking, speaking, or toileting | 3 (30.0%) |
| Decreased emotional expression^*^ | Described after treatment as being able to show the necessary emotions when needed | 1 (10.0%) |
| Impacted self-image^*^ | *Described after treatment as having the appearance of a normal child, with a lack of swelling in various parts of the body and lesions* | 1 (10.0%) |
| Impaired perception/‌reaction^*^ | Described after treatment as improved perceptions and reactions while playing | 1 (10.0%) |
| Emotional impacts | | |
| Sadness | Described by patients as feeling sad due to CHAPLE disease and its symptoms (e.g., stomachache, nausea) or concerns about their mother, who has previously lost a child to the disease  Described by caregivers as being sad due to the inability to participate in activities, being mocked by other children, or crying due to symptoms | 7 (70.0%) |
| Anger | Caregivers described anger as the child getting angry more easily or being more short-tempered due to the condition or feeling mad due to others trying to avoid them and/or when they are unable to express feelings | 2 (20.0%) |
| Fear | A patient and a caregiver described fear due to the prospect of experiencing abdominal pain that does not end despite vomiting | 3 (30.0%) |
| Annoyance | A patient described annoyance as feeling irritated at being unable to go to school, resulting in a lack of understanding in class, or feeling irritated when going out | 1 (10.0%) |
| Boredom | A patient described boredom due to the regular occurrence of symptoms and the hindrance it causes in their life | 1 (10.0%) |
| Embarrassment | A caregiver described the child feeling embarrassment when they had an accident and had a bowel movement accident that resulted in the child not wanting to go out | 2 (20.0%) |
| Feeling different from peers | A caregiver described the child feeling like they cannot be like other kids due to pain and inability to eat like others | 1 (10.0%) |
| Feeling self-conscious | A patient described being bothered by the condition due to the inability to run and have fun like other children | 2 (20.0%) |
| Worry/using extra caution during physical activities | Described as being extra careful about affecting post-surgical incisions and being more nervous/short-tempered | 3 (30.0%) |
| Healthcare utilization | | |
| Hospitalization | Described by a patient as spending time at the hospital getting shots and vaccines and having blood taken  Described by caregivers as trips to the hospital, spending longer terms at intensive care, or using emergency services up to twice a day due to symptoms such as vomiting and fever | 7 (70.0% |
| Inability to attend rehabilitation | No description was provided by the caregiver about the child’s inability to attend rehabilitation | 1 (10.0%) |
| Need for doctor visits | A caregiver described the need to take the child to the doctor due to abdominal pain and vomiting | 1 (10.0%) |
| Household chores/responsibilities | | |
| Difficulty completing housework | A patient described difficulty completing housework as being unable to help their mother as much as they previously were able to, due to fatigue | 3 (30.0%) |
| Leisure impacts | | |
| Difficulty engaging in play activities | Described by patients as being unable to ride bikes, play ball, play with siblings; having to quit playing games; or being unable to go out and play with friends when experiencing stomach pain, nausea, headaches, bloating, or diarrhea  Described by caregivers as children being unable to play, either alone or with other children, due to symptoms such as lack of strength, stomachache, and facial edema/swelling | 8 (80.0%) |
| Inability to participate in sedentary leisure activities | Described by patients and caregivers as being unable to continue or engage in coloring, painting, or watching television due to stomachache | 3 (30.0%) |
| Inability to travel | Described by caregivers as the family’s inability to take trips, engage in activities, or go to other places due to symptoms experienced by their children | 2 (20.0%) |
| Limited ability to participate in active leisure activities | Described by patients as the inability to jump rope or the inability to play sports as being unable to play football, basketball, or other sports due to stomach pain or headache | 3 (30.0%) |
| Decreased desire to go outside | A caregiver described the decreased desire to go outside as the child refusing to go outside due to stomachache, diarrhea, and vomiting | 1 (10.0%) |
| Physical impacts | | |
| Feeling exhausted/‌weak | Described by patients as being unable to go about with the rest of the day due to diarrhea or severe abdominal pain  Described by caregivers as becoming a different person, accompanied by feelings of tiredness, weakness, and restlessness, as well as appearing pale, dehydrated, or with sunken eyes | 6 (60.0%) |
| Inability to run | Described by patients as being unable to run fast or at all due to symptoms (e.g., stomachache worsening when running)  A caregiver described inability to run as the child not running during physical education class due to concerns about stomach pain | 5 (50.0%) |
| Impaired ability to walk | A patient described impaired ability to walk as being due to pain throughout the body  Described by caregivers as the child being unable to walk due to delayed development, or needing to stop walking and use a stroller when fatigued | 4 (40.0%) |
| Impaired growth | Described by a patient and caregivers as being unable to grow due to CHAPLE disease, or as the child being smaller and paler as they are limited in what they can eat | 7 (70.0%) |
| Loss of appetite | A caregiver described loss of appetite as the child not having any desire to eat until the evening. No further description was provided by the patient | 4 (40.0%) |
| Difficulty chewing and swallowing | A caregiver described difficulty chewing and swallowing as difficulty eating due to underdeveloped muscles | 1 (10.0%) |
| Inability to stand | A caregiver reports that their child cannot stand; no further description was provided | 1 (10.0%) |
| Increased need for hydration | A caregiver described increased hydration as the need to drink more water to remove edema/swelling | 1 (10.0%) |
| Impaired physical function^*^ | Described after treatment as lack of difficulty doing physical activities such as running, walking, playing, and exercising | 1 (10.0%) |
| Impaired vision^*^ | Described as swelling of the eye that causes it to become partially shut, reducing the field of vision | 2 (20.0%) |
| Inability to sit^*^ | Described as being unable to sit down straight and establish balance | 1 (10.0%) |
| Muscle weakness^*^ | Described by caregiver as weak bones, and the inability to lift heavy objects, or to jump up and down. When muscles were weak, patients reported having almost no strength  Described by patient as the feeling of being stronger and having more energy, and being observed as not experiencing weakness and nausea | 2 (20.0%) |
| Restlessness^*^ | Described as a feeling stemming from swelling in eyelids and feet. Swollen eyelids and swollen feet caused nausea and weakness and contributed to restlessness | 1 (10.0%) |
| Other family/friend relationships | | |
| Impacts to family relationships | Described by caregivers as being more sensitive to the afflicted child and giving the child with CHAPLE disease objects from other children due to the illness. Some caregivers reported other children displayed resentment due to the child with CHAPLE disease receiving more attention; one caregiver reported siblings taking care of one another | 4 (40.0%) |
| School impacts | | |
| Missing school | Described by patients and caregivers as missing significant amounts of school due to stomachache, nausea, diarrhea, swelling, or hospitalizations due to symptoms, as well as due to restricted diet and distance from home | 9 (90.0%) |
| Interference with schoolwork | A patient described interference with schoolwork as the inability to get assignments in on time due to stomach pain  A caregiver described interference with schoolwork as the child not understanding homework due to the inability to go to class. This caregiver also described the child having to take exams later when they miss school due to symptoms | 2 (20.0%) |
| Need for additional teacher care | A caregiver described need for additional teacher care as the child receiving additional attention from their teacher in order to keep them safe | 1 (10.0%) |
| Inability to enroll in school activities | A caregiver described inability to enroll in school activities as the children not being included in activities in case of a disease-related accident | 1 (10.0%) |
| Inability to study | A patient described inability to study as an inability to get prepared for their university exam due to the disorder | 1 (10.0%) |
| Impaired performance at school^*^ | Described as falling behind one’s peers and classes due to lack of ability to study and/or attend school | 2 (20.0%) |
| Loss of interest at school^*^ | Described after treatment as loss of desire and enthusiasm to catch up in school due to losing so many years of schooling because of patient’s disease | 1 (10.0%) |
| Sleep impacts | | |
| Sleep disruptions | Described by patients and caregivers as patients being unable to sleep or being woken up by pain due to symptoms (e.g., diarrhea), or the inability for patients to sleep without medication | 10 (100.0%) |
| Sleepiness | A caregiver described sleepiness as the child becoming sleepy when experiencing pain, though the pain may prevent the ability to fall asleep | 2 (20.0%) |
| Difficulty falling asleep^*^ | Described as being unable to fall asleep due to stomach pain | 1 (10.0%) |
| Social impacts | | |
| Impaired ability to socialize with other children | Described by a patient as the inability to meet up with friends for planned activities due to symptoms  Described by caregivers as a refusal to socialize with other children due to past bullying, not seeing friends due to missing school, or an inability to make friends due to the condition | 6 (60.0%) |
| Interference with family activities | A participant described interference with family activities as an inability to spend time with family or having family activities postponed due to hospitalizations  Described by caregivers as the child’s inability to spend time visiting with relatives due to CHAPLE disease-related concerns (e.g., facial edema/swelling, perceived aggression) | 6 (60.0%) |
| Bullied by friends/other children | Described by patients and a caregiver as being teased, friends accusing them of faking the illness and instructing others to do the same, or as other children making fun of the patient due to CHAPLE disease | 3 (30.0%) |
| Social isolation | A patient described social isolation as the disease causing them to want to spend life alone, as well as causing a lack of friends and socializing  Described by caregivers as the child acting withdrawn (e.g., going into a shell, ignoring requests to play) or not wanting to engage in activities with others due to symptoms | 6 (60.0%) |
| Impacted classmate relationships | A patient described impacted classmate relationships as classmates not letting the patient into groups due to the chance that the patient would be absent during the day they present their work | 2 (20.0%) |
| Impaired social development^*^ | Described as being unable to socialize with others due to rejections because of symptoms or recurrent hospitalization, causing lack of relationships with others that affect development | 3 (30.0%) |
| Social perception of sickness^*^ | Described as not being allowed to do activities, such as lift items, because others assume the patient is too sick to do so | 1 (10.0%) |

^*^Sixteen impact concepts across 11 conceptual domains were not reported at screening but were reported at W24 to have been experienced by patients prior to treatment.
